# O13 A case of chronic recurrent multifocal osteomyelitis

**DOI:** 10.1093/rap/rkab067.012

**Published:** 2021-10-19

**Authors:** Jessica M Weightman, Geetha L Janakiraman

**Affiliations:** James Cook University Hospital, Middlesbrough, United Kingdom

## Abstract

**Case report - Introduction:**

We present the case of a young adult female who presented to rheumatology with persistent arthralgia of her knees despite disease-modifying anti-rheumatic drugs (DMARDs) for seronegative inflammatory arthritis. Her magnetic resonance imaging (MRI) of her knees demonstrated bone lesions compatible with chronic recurrent multifocal osteomyelitis (CRMO). CRMO is a rare autoinflammatory condition of the bone, typically affecting younger female patients. It is characterised by the presence of multifocal inflammatory bone lesions, often affecting the metaphyses of long bones. It can present with localised pain and swelling, and such as in this case, joint swelling.

**Case report - Case description:**

An 18-year-old female patient presented to rheumatology in October 2019 with a 4-week history of left knee monoarthritis.

Due to a history of a preceding upper respiratory tract infection, oral non-steroidal anti-inflammatories (NSAIDs) were commenced for possible reactive arthritis. She had no history of seronegative features of psoriasis, uveitis, inflammatory bowel disease, pustulosis or acne.

Over the next 4 weeks, she experienced intermittent episodic bilateral knee effusions associated with elevated inflammatory markers. C-reactive protein (CRP) peaked at 232mg/L and erythrocyte sediment rate (ESR) at 57mm/1hr. Rheumatoid factor, anti-cyclic citrullinated peptide (CCP) antibodies and antinuclear antibody (ANA) were negative. Plain X-rays of the knees were normal.  MRI of the right knee and hip in October 2019 were reported as normal. An ultrasound scan of the right knee in December 2019 between episodes of swelling, was normal.

She continued to experience persistent knee and right hip arthralgia, with stiffness and limitation of knee flexion. DMARD treatment was commenced in December 2019 due to intermittent synovitis of the knees. Inflammation in the knees persisted despite treatment with oral Methotrexate. The additional Sulfasalazine was discontinued due to neutropaenia. Etanercept (Benepali) was commenced in November 2020 with good clinical and biochemical response. However, despite resolution of knee effusions, knee arthralgia persisted with stiffness and limited knee flexion. Repeat MRI imaging revealed multiple areas of hypointense signal surrounding central areas of hyperintense signal. These are characteristic of CRMO. On review of the initial right knee MRI from October 2019, it was noted that subtle lesions were visible. Bone turnover markers were checked; beta-crosslaps was 573ng/L and type 1 procollagen peptide was 59.7ug/L. The patient has now begun a 1-year treatment course of IV pamidronate in addition to continuing her methotrexate and Benepali.

**Case report - Discussion:**

CRMO is a rare condition, affecting approximately 4 per million. It typically affects children, more commonly females. Although the most common presenting symptoms are pain and local tenderness, patients may also report warmth and localised swelling. Systemic features can occur, albeit rarely. Although it can affect multiple sites, including the spine, the metaphyses of long bones are commonly involved.

CRMO is a non-infectious autoinflammatory bone disorder, thought to be due to abnormal production of pro- and anti-inflammatory cytokines, which in turn affect the activation of osteoclasts within bone. Although plain X-rays may be normal, they may show evidence of osteolytic lesions or sclerosis later in the disease process. MRI is the preferred imaging modality and can demonstrate areas of bone marrow oedema and periostitis.

The MRIs of this patient showed multiple large geographic lesions bilaterally demonstrating hypointense signal peripherally with central areas of hyperintense (high) signal. Both the epiphyses and metaphyses of the long bones were affected, which is characteristic of CRMO.  Similar appearances could be seen in bone infarcts, for example in sickle cell disease; however, these would usually only affect the metaphyses of bone. Although the previous imaging was reported to be unremarkable, some subtle lesions can be seen.

This patient’s isotope bone scan revealed non-specific uptake in the knees and shoulders, rather than localised areas of uptake which are typical in CRMO. A bone biopsy can differentiate CRMO from important differentials such as infective osteomyelitis and malignancy. In this patient, a bone biopsy was discussed but not undertaken as there was widespread involvement with no clear satellite lesion.

Management of CRMO encompasses a variety of treatments, including NSAIDs, steroids, DMARDs and bisphosphonates largely based upon smaller trials. Due to ongoing symptoms despite conventional synthetic and biologic DMARDs, in this patient IV pamidronate treatment was commenced.

**Case report - Key learning points:**

This case highlights the importance of considering CRMO in young adult patients with seronegative arthritis who do not respond to standard therapy.

There were no identifiable features of bone inflammation on either plain X-rays or knee ultrasound and no reported features on the initial MRI of the right knee. This case reinforces the need for multidisciplinary team discussion with an experienced specialist musculoskeletal radiologist, especially when symptoms are discordant from the clinical examination findings, posing diagnostic uncertainty. In this case, it transpired that evidence of CRMO could be seen on the initial MRI which was taken approximately 18 months before the diagnosis was made.

Given that this patient had active bone inflammation despite treatment with conventional synthetic and biologic DMARDs, she was commenced on intravenous bisphosphonates which inhibit osteoclastic activity and can reduce inflammatory cytokine production. Bisphosphonates can cross the placenta and potentially impair normal bone development in utero.  This can pose a management challenge in young adult females of child-bearing age.

Pamidronate has been shown to be useful in cases of SAPHO (synovitis, acne, pustulosis, hyperostosis, osteitis); a related condition where non-infectious osteomyelitis is associated with dermatological manifestations of acne and pustulosis. Treatment response can be monitored by clinical improvement and by biochemical markers of bone turnover, such as beta-crosslaps and type 1 procollagen peptide (P1NP).

In this case, the patient was found to be vitamin D deplete and required replacement therapy prior to pamidronate therapy. After initiating treatment, bone turnover markers have reduced although arthralgia persists. A 1-year treatment course has been planned with a repeat MRI to reassess radiological response at that time.

